# Intestinal Parasites of Zoonotic Significance in Human and Domestic Animals in a Rural Setting in Nepal

**DOI:** 10.1002/vms3.70728

**Published:** 2025-12-11

**Authors:** Prince Rai, Tirth Raj Ghimire

**Affiliations:** ^1^ Department of Zoology Tri‐Chandra Multiple Campus Tribhuvan University Kathmandu Nepal; ^2^ Central Department of Zoology Tribhuvan University Kathmandu Nepal

**Keywords:** *Cryptosporidium*, modified acid‐fast staining, one health, Solukhumbu, zoonosis

## Abstract

Intestinal parasitic (IP) species with zoonotic significance are major public health issues, mostly prevalent in developing countries, leading to high morbidity and mortality. The present study aimed to assess the prevalence of IP species in humans and domestic animals, compare and contrast the zoonotic potentialities and investigate the associated factors in the Solukhumbu District, Nepal. The fresh faecal samples from humans (*N* = 200), cattle (*N* = 20), dogs (*N* = 20) and pigs (*N* = 20) were collected using a non‐invasive purposive sampling method, preserved in 2.5% potassium dichromate, and examined by direct wet mount, flotation and modified acid‐fast techniques. Sociodemographic data were obtained through a structured questionnaire, interviews and focal group discussions. The overall prevalence rate of IP species in all hosts was recorded as 70.77% (184/260), with infection rates of 65.5% in humans, 100% in cattle and pigs and 65% in dogs, with a total of 23 species. *Cryptosporidium* spp., *Balantidium coli*, *Ascaris* spp., taeniid and Acanthocephala spp. were shared by all hosts. Taeniid (18.5%) in humans, *Entamoeba* spp. (85%) in cattle, Strongyle (70%) in pigs and *Cryptosporidium* spp. (30%) in dogs were the most prevalent species. Significant associations were observed between a few IP species and participants’ characteristics like education status, drug intake history, occupation type, feeding habit and disease checkup priorities (*p* < 0.05). These findings reinforce the need to consider deworming campaigns for both humans and domestic animals. Further, one‐health approaches involving extensive datasets of faecal samples from animals and humans living nearby and environmental samples would address zoonoses more effectively.

## Summary

- Our study highlighted the possible zoonoses between humans and domestic animals, sharing a complex rural agricultural landscape where five different zoonotic intestinal parasitic species, like *Cryptosporidium* spp., *Balantidium coli*, *Ascaris* spp., taeniid and Acanthocephala spp., were common in all four hosts.
- It is recommended that policymakers should run immediate, effective deworming campaigns for both the human and the domestic animals, and implementing one health strategies is essential to reduce zoonotic transmission of such shared parasites.

## Graphical abstract

Intestinal parasites are shared among humans and domestic animals.

Rural setting is critical to enhance parasite zoonosis in Nepal.

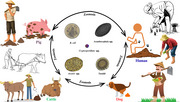

## 1 Introduction

Intestinal parasitic infections (IPIs) are a vital epidemiological concern, causing illness and fatality globally (Ahmed 2023). The factsheet prepared by the World Health Organization (WHO) revealed that approximately 24% of the worldwide population is affected by IPIs (WHO 2023). IPIs are considered neglected tropical diseases aimed to be eradicated by 2030 as part of the Sustainable Development Goals (SDGs) (WHO 2025).

Importantly, the most common human intestinal parasitic (IP) species responsible for IPIs among the people in various landscapes of the Hindu‐Kush Himalayan regions, including Nepal, are *Entamoeba coli*, *Entamoeba histolytica*, *Cryptosporidium* spp., *Giardia*, *Taenia* spp., *Ascaris lumbricoides*, *Trichuris trichiura*, hookworm, *Strongyloides stercoralis* and *Hymenolepis nana* (Ghimire et al. 2020; Feng et al. 2021; Pawestri et al. 2021; Karim et al. 2024; Yadav et al. 2024; Chaudhary and Ghimire 2025). People living in rural areas of developing countries are typically at a higher risk of IPIs (Karim et al. 2024). This increased risk is attributed to various risk factors like illiteracy, poor sanitation practices, contaminated sources of drinking water, inadequate food hygiene, close animal contact, lack of antiparasitic treatment and seasonal variations (Adhikari et al. 2021b; Sebaa et al. 2021; Nath et al. 2022; Gautam et al. 2024; Bhandari et al. 2025). Due to the parasitic burden in the human body, one can suffer from asymptomatic, acute to severe infections causing fatal diarrhoea, or even blood deficiency leading to anaemia, and finally to death (Ahmed 2023). IPIs, along with other metabolic diseases, also seem to favour comorbidities in many individuals (Oliveira et al. 2022). Opportunistic IP species such as *Cryptosporidium* spp., *Cystoisospora belli*, *Microsporidia* spp. and *S. stercoralis* can impair the immunocompromised host's immunity through immunomodulation, primarily by interfering with gut‐associated lymphoid tissues (GALTs) (Olatunde et al. 2023; Sharma and Khurana 2025), suggesting the sources of these parasites as an important public health factor around the world.

Around 66% of Nepal's total population is engaged in agriculture (FAO 2025). Thus, the majority of Nepalese people depend on crop and vegetable production, as well as livestock farming, to meet their family needs, contributing to the country's sustainable development. Agriculture accounts for 21.9% of Nepal's national gross domestic product (GDP) (The World Bank Group 2025). Domestic animals like cattle, buffalo, pigs, goats and dogs have always been close to humans. Hence, these animals under captive, semi‐captive, or free‐ranging conditions near humans may risk zoonosis due to usual contact with zoonotically important parasites. Some IP species like *Entamoeba* sp., *Giardia* sp., *Sarcocystis* spp., *Balantidium coli*, hookworm, taeniid, *Toxocara canis*, *Cryptosporidium* spp., *Toxoplasma* and *Fasciola* spp. transmit zoonotic diseases to humans (Chaudhary et al. 2023; Sukupayo and Tamang 2023; Adhikari et al. 2025). The World Organization for Animal Health (OIE) and WHO estimated a large proportion of worldwide human infectious illnesses (60%) to be of animal origin, highlighting the emerging impact of zoonoses on public health (Haider et al. 2020). Considering the emergence of parasitic zoonosis worldwide, the Zoonoses Control Program of Nepal has also categorized six Prioritized Zoonotic Diseases: leptospirosis, avian influenza, brucellosis, toxoplasmosis, taeniasis or cysticercosis or neurocysticercosis and hydatidosis, out of which the latter two are critical IPIs with epidemic potentialities (EDCD 2025). Notably, these parasitic diseases are primarily diagnosed in people who live near domestic animals involved in farming.

Therefore, the current study was undertaken in Mapya Dudhakoshi Rural Municipality (MDRM) in a rural setting where many people practice agriculture and live in proximity to domestic animals. Hypothetically, there may be the transmission of IPIs between humans and domestic animals. However, no study has been conducted regarding this aspect in this MDRM. On that account, the study was conducted by examining the IP species in faeces samples of humans and nearby domestic animals like cattle, pigs and dogs. In addition, we aimed to analyse how IP zoonosis might exist in this rural setting in a complex agricultural landscape for the first time in Nepal. Previous studies on zoonosis have lacked explaining the causal association of IP species in leading to infection in humans and have not been thoroughly analysed in Nepal. Thus, this study aimed to find out the prevalence, intensity and diversity of parasites and assess their potential risk factors among both people and domestic animals of MDRM, Solukhumbu, Nepal.

## 2 Materials and Methods

### 2.1 Study Area

**FIGURE 1:**
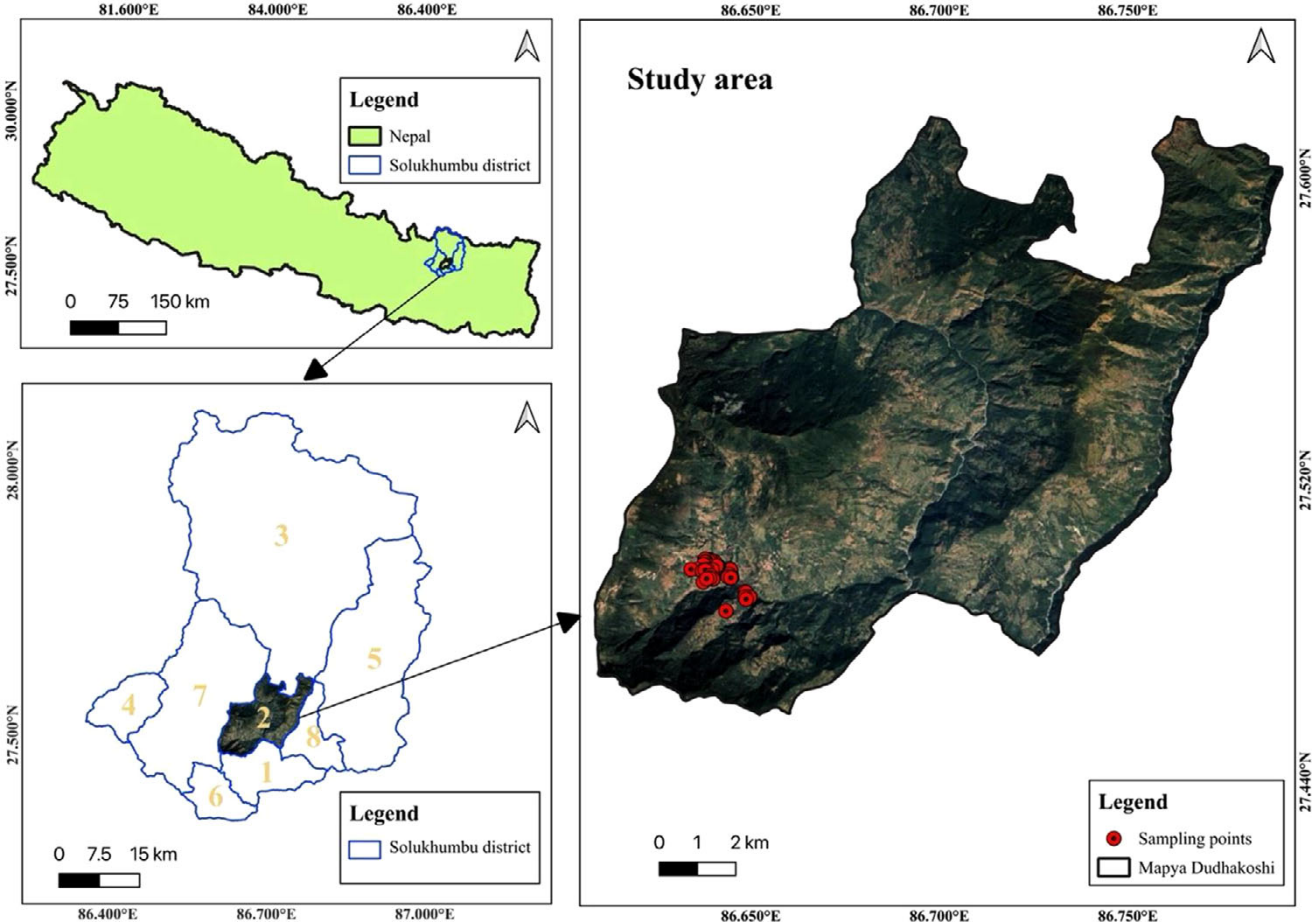
Map of Nepal indicating the study area, MDRM, Solukhumbu, Nepal (base map imagery adapted from Google Satellite via QGIS).

The study was conducted in the MDRM of Solukhumbu district of Nepal, that is, situated in the mountainous region of northeastern Nepal, with an area of 3312 sq km and a population of 104,851 (Figure 1). MDRM has an area of 167.67 sq. km and a total population of 12,648 (CBS 2022). It lies in the mid‐hill region called the Solu region in the lower parts of the Solukhumbu district. The study was conducted in Ward Number 5, which has an area of 35.76 sq. km and a total population of 2218, while the total population of people equal to or above 18 years is 1929 (ECN 2022). It is inhabited by the most diverse ethnic groups, like Rai, Sherpa, Tamang, Chhetri and Bishwokarma. People living in this area are more likely to engage in agricultural practices and domestic animal farming, where people rear cattle, buffalo, goats, pigs and dogs.

### 2.2 Study Design

The cross‐sectional study was designed to assess the prevalence of IP species in the people and domestic animals of MDRM by collecting stool samples and examining them, along with conducting a structured questionnaire survey about the risk factors of IPIs in the studied population from January 2024 to April 2024.

### 2.3 Sample Selection and Sampling

#### 2.3.1 Sample From Humans

A total of 200 human participants were purposively selected to represent diverse sociodemographic characteristics, including age, sex and occupation. Data were collected using structured questionnaires (*N* = 200) and focal group discussions among five individuals of varied professions (student, health worker, politician, farmer and housewife). Verbal and written consent were obtained before participation. Participants were given sterile, labelled vials and application sticks with clear instructions regarding stool sample collection. About 20 g of fresh stool samples were collected from each targeted person in sampled households. The stool sample collection technique was a non‐invasive type. Each vial was labelled with the participant's name, code number, date and time of collection.

#### 2.3.2 Sample From Domestic Animals

Additionally, 60 fresh faecal samples from three domestic animals (20 each from cattle, dogs and pigs) were purposively collected from the same households of the selected human participants who had reared any one of them. We ensured to collect those samples carefully to avoid mixing with any contamination of faeces from other hosts.

We followed the domestic dogs carefully within their premises and collected their fresh faecal samples that fell on the ground immediately after defecation.

In contrast, we collected the fresh swine and cattle faecal samples that fell on the ground just after defecation from the pigsty and cowshed, respectively.

#### 2.3.3 Inclusion Criteria of Humans and Domestic Animals

Only individuals who were mentally and physically healthy and over 18 years old, based on family members' information, were interviewed. Only individuals who wanted to participate in the study were involved. And samples were collected exclusively from healthy animals.

### 2.4 Exclusion Criteria of Humans and Domestic Animals

Individuals less than 18 years old and those with mental and physical disabilities, based on the family members' information, were excluded from the study. Individuals who did not wish to participate in the study were excluded. Physically impaired domestic animals were not involved in the study.

### 2.5 Sample Preservation and Transportation

The collected stool samples were preserved in 2.5% K_2_Cr_2_O_7_ and transported to the Research Laboratory of the Department of Zoology, Tri‐Chandra Multiple Campus, for further investigation of cysts, trophozoites, eggs and larvae of IP species.

### 2.6 Laboratory Processing and Examination

The samples were examined macroscopically with the naked eye to check for the presence of blood, mucus and worms. Before microscopic examination, each sample was homogenized by vortex. The following laboratory processes were performed based on techniques discussed in the literature (Adhikari et al. 2021b; Adhikari et al. 2023; Adhikari et al. 2025; Chaudhary and Ghimire 2025).

#### 2.6.1 Direct Mount Technique

Each faecal sample was observed via direct wet mount by three times consecutively. For this, the faecal sample preserved in the vial was mixed gently with the help of an applicator stick. A drop of the sample was kept on an uncontaminated glass slide, one without iodine, whereas the other was stained with Lugol's iodine, both covered by a cover slip. Finally, the slides were examined under a microscope at 100x and 400x magnifications.

#### 2.6.2 Saturated Flotation Technique

Each faecal sample was observed via floatation one time. For this, about 2 g of the stool sample was mixed with 12 mL of 45% w/v NaCl in a test tube and vortexed. Then, the tube was left undisturbed for 5 min, covering its tip with a coverslip. Eventually, the coverslip was kept on an uncontaminated glass slide and observed under 100x and 400x magnifications.

#### 2.6.3 Modified Acid‐Fast Staining Technique

All the coccidian‐positive samples were processed for modified acid‐fast staining. For this, a thin smear of the coccidian‐positive faecal sample was prepared and allowed to air dry for a while at room temperature. Air‐dried smear was then heat‐fixed in absolute methanol for 2 min, followed by the staining process with carbol fuchsin for 10–15 min. The slide was then cooled and carefully washed under running tap water for 1 min. Afterward, the smear was destained using acid alcohol and again rinsed with water for 1 min. At last, the smear was stained with malachite green and air‐dried. Eventually, the dry slide using immersion oil was examined under a magnification of 1000X.

#### 2.6.4 Observation and Identification

All stages of parasite cysts, trophozoites, oocysts, eggs, and larvae were examined using the compound microscope (Olympus CX23), and identified based on morphometric pictures provided by the Centers for Disease Control and Prevention (CDC 2019) and previously published literature (Adhikari et al. 2021b; Adhikari et al. 2023) and books (Soulsby 2012; Zajac and Conboy 2012). Also, the photographs were identified by the researchers and experts.

### 2.7 Data Analysis

The data were analysed using MS Excel 2010 with tables and figures. Multivariate analysis using the chi‐squared test by trend and bivariate analysis using Fisher's exact test values at different degrees of freedom were performed using GraphPad Prism (Prism 5 for Windows Version 5.00, 2007). The statistical values were considered significant at a 95% confidence interval (5% level of significance), that is, *p* < 0.05. A four‐oval flower model Venn diagram was created to highlight the common zoonotic species shared between the four hosts (Someka.net). The parasite intensity was assessed based on the number of parasites observed per field view under the microscope. A count of one to three parasites per field (light infection) was recorded as +, 4–10 parasites per field (moderate infection) ++ and more than 10 parasites per field (heavy infection) +++, using a 10x objective (100x magnification) for helminths and the largest ciliate protozoan *B. coli* and 40x objective (400x magnification) for all other protozoa based on the methodology given in literature (Sood 1994; Acharya et al. 2025).

### 2.8 Ethical Approval

Ethical permission was granted by the Institutional Research Committee, Institute of Science and Technology, Tribhuvan University (Ref. Number: 167/080/081) and permission for sample collection was approved by the Ward Number 5 Office, MDRM, Solukhumbu, Nepal (Dispatch Number: 209) to conduct the research.

## 3 Results

### 3.1 General Prevalence (%) of IP Species in Humans and Domestic Animals

The overall prevalence rate of 70.77% was recorded in all four hosts. The cattle and pigs had a 100% prevalence rate of IP species, while humans and dogs had 65.5% and 65% prevalence rates, respectively. Out of 23 IP species recorded, 19 IP species were of zoonotic significance. The prevalence rates of IP species like *Entamoeba* spp., *E. histolytica*, *B. coli*, *Eimeria* spp., *Cystoisospora* sp., *Sarcocystis* sp., *Neospora caninum*, *Dipylidium caninum*, *T. canis*, hookworm, Strongyle, *Trichuris* sp. and Acanthocephala spp. showed a statistically significant difference among various hosts (*p* < 0.05) (Table 1) (Figure 2).

**TABLE 1:** General prevalence (%) of IP species in humans and domestic animals.

Protozoan parasites	Humans (*n* = 200)	Cattle (*n* = 20)	Pigs (*n* = 20)	Dogs (*n* = 20)	Total (*N* = 260)	*χ* ^2^ value	*p*‐value
Sarcodina							
*Entamoeba* spp.^a^	30 (15)	17 (85)	11 (55)	1 (5)	59 (22.69)	66.47	*p* < 0.05
*Entamoeba histolytica* ^a^	18 (9)	**—**	—	—	18 (6.92)	9.607	*p* < 0.05
*Endolimax* spp.^a^	9 (4.5)	**—**	2 (10)	—	11 (4.23)	0.1274	ns
*Iodamoeba* spp.^a^	7 (3.5)	1 (5)	1 (5)	—	9 (3.46)	0.1774	ns
*Blastocystis* spp.^a^	6 (3)	2 (10)	—	—	8 (3.08)	0.4275	ns
Flagellata							
*Giardia* ^a^	5 (2.5)	—	—	—	5 (1.92)	1.257	ns
Ciliata							
*Balantidium coli* ^a^	8 (4)	4 (20)	11 (55)	1 (5)	24 (9.23)	17.07	*p* < 0.05
Apicomplexa							
*Cryptosporidium* spp.^a^	36 (18)	4 (20)	5 (25)	6 (30)	51 (19.62)	2.022	ns
*Cyclospora* spp.^a^	18 (9)	5 (25)	1 (5)	1 (5)	25 (9.62)	0.1212	ns
*Eimeria* spp.	**—**	**—**	8 (40)	3 (15)	11 (4.23)	43.61	*p* < 0.05
*Cystoisospora* sp.	**—**	**—**	—	5 (25)	5 (1.92)	38.03	*p* < 0.05
*Sarcocystis* sp.^a^	**—**	**—**	—	1 (5)	1 (0.38)	7.488	*p* < 0.05
*Neospora caninum*	**—**	**—**	—	2 (10)	2 (0.77)	15.03	*p* < 0.05
Total protozoan	**87 (43.5)**	**18 (90)**	**18 (90)**	**12 (60)**	**135 (51.92)**	**13.68**	*p* < 0.05

Helminth parasites	Humans (*n* = 200)	Cattle (*n* = 20)	Pigs (*n* = 20)	Dogs (*n* = 20)	Total (*N* = 260)	*χ* ^2^ value	*p*‐value
Cestoda							
Taeniid^a^	37 (18.5)	7 (35)	6 (30)	5 (25)	55 (21.15)	1.981	ns
*Dipylidium caninum* ^a^	**—**	**—**	—	1 (5)	1 (0.38)	7.488	*p* < 0.05
Nematoda							
*Ascaris* spp.^a^	32 (16)	9 (45)	6 (30)	3 (15)	50 (19.23)	1.374	ns
*Toxocara canis* ^a^	**—**	—	—	3 (15)	3 (1.15)	22.64	*p* < 0.05
*Strongyloides* spp.^a^	18 (9)	—	5 (25)	1 (5)	24 (9.23)	0.1965	ns
Hookworm^a^	4 (2)	—	4 (20)	4 (20)	12 (4.62)	21.15	*p* < 0.05
Strongyle^a^	3 (1.5)	—	14 (70)	—	17 (6.54)	29.59	*p* < 0.05
*Oxyuris* sp.	**—**	1 (5)	—	—	1 (0.38)	0.3369	ns
*Trichuris* sp.^a^	**—**	**—**	2 (10)	—	2 (0.77)	5.522	*p* < 0.05
Acanthocephala							
Acanthocephala spp.^a^	1 (0.5)	1 (5)	3 (15)	1 (5)	6 (2.31)	10.33	*p* < 0.05
Total helminth	**74 (37)**	**12 (60)**	**17 (85)**	**10 (50)**	**113 (43.46)**	**10.72**	*p* < 0.05
Total parasites	**131 (65.5)**	**20 (100)**	**20 (100)**	**13 (65)**	**184 (70.77)**	**4.265**	*p* < 0.05

*Note*: ^a^The zoonotic parasites.

Abbreviation: ns, not significant.

FIGURE 2(a) *Cryptosporidium* sp. (Dogs), (b) *Cyclospora* sp. (Cattle), (c) *Giardia* (Humans) (d) *Entamoeba coli* (Humans), (e) *Entamoeba histolytica* (Humans), (f) *Balantidium coli* (Pigs), (g) *Blastocystis hominis* (Humans), (h) *Cystoisospora* sp. (Dogs), (i) *Eimeria* sp. (Dogs), (j) *Eimeria* sp. (Pigs), (k) *Ascaris* sp. (Humans), (l) *Ascaris* sp. (Cattle), (m) *Ascaris* sp. (Dogs), (n) *Toxocara canis* (Dogs), (o) Taeniid (Dogs), (p) Taeniid (Humans), (q) Taeniid (Cattle), (r) *Strongyloides* sp. (Pigs), (s) *Trichuris* (Pigs), (t) Hookworm (Dogs), (u) Hookworm (Pigs).

**Figure:**
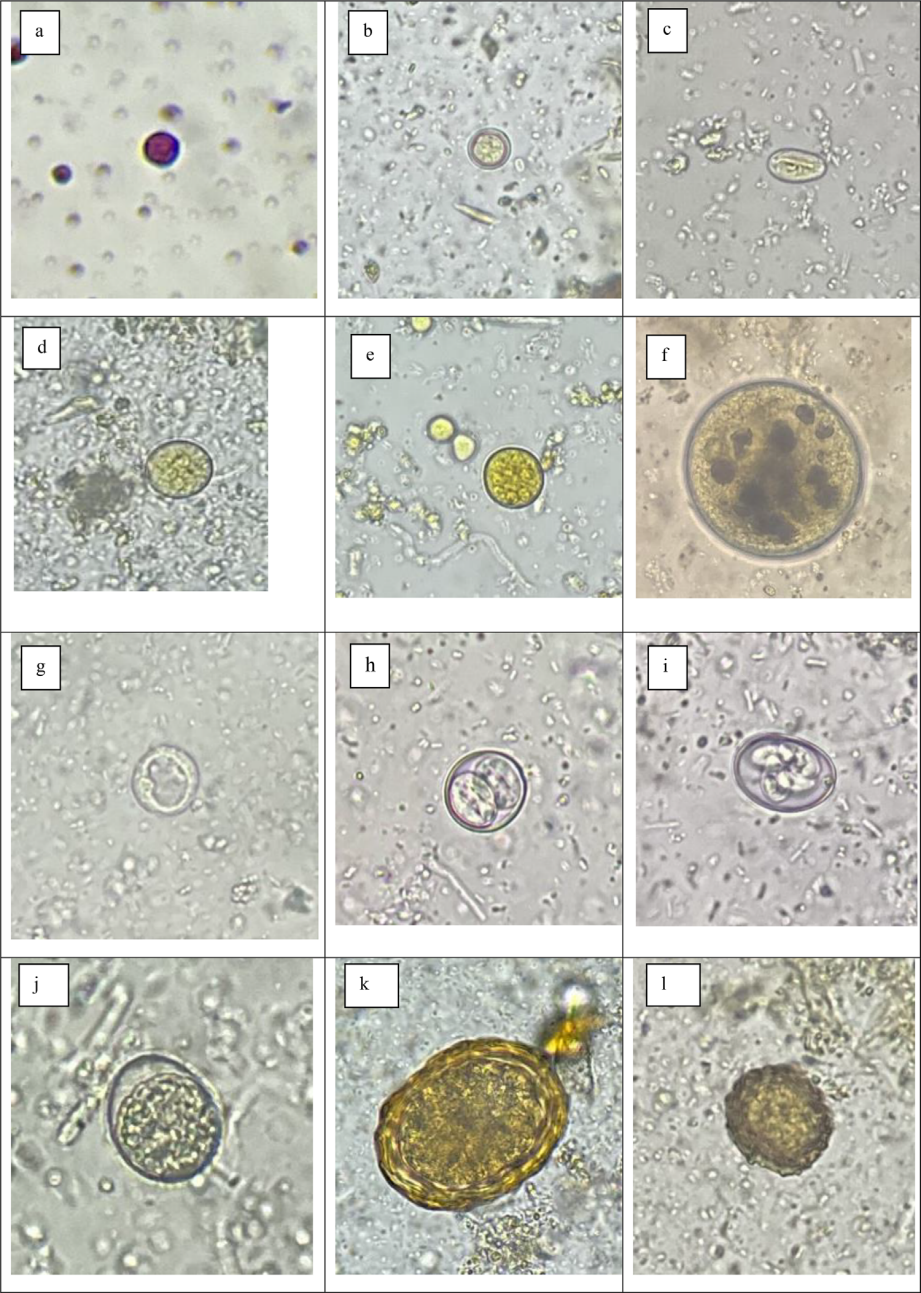

**Figure:**
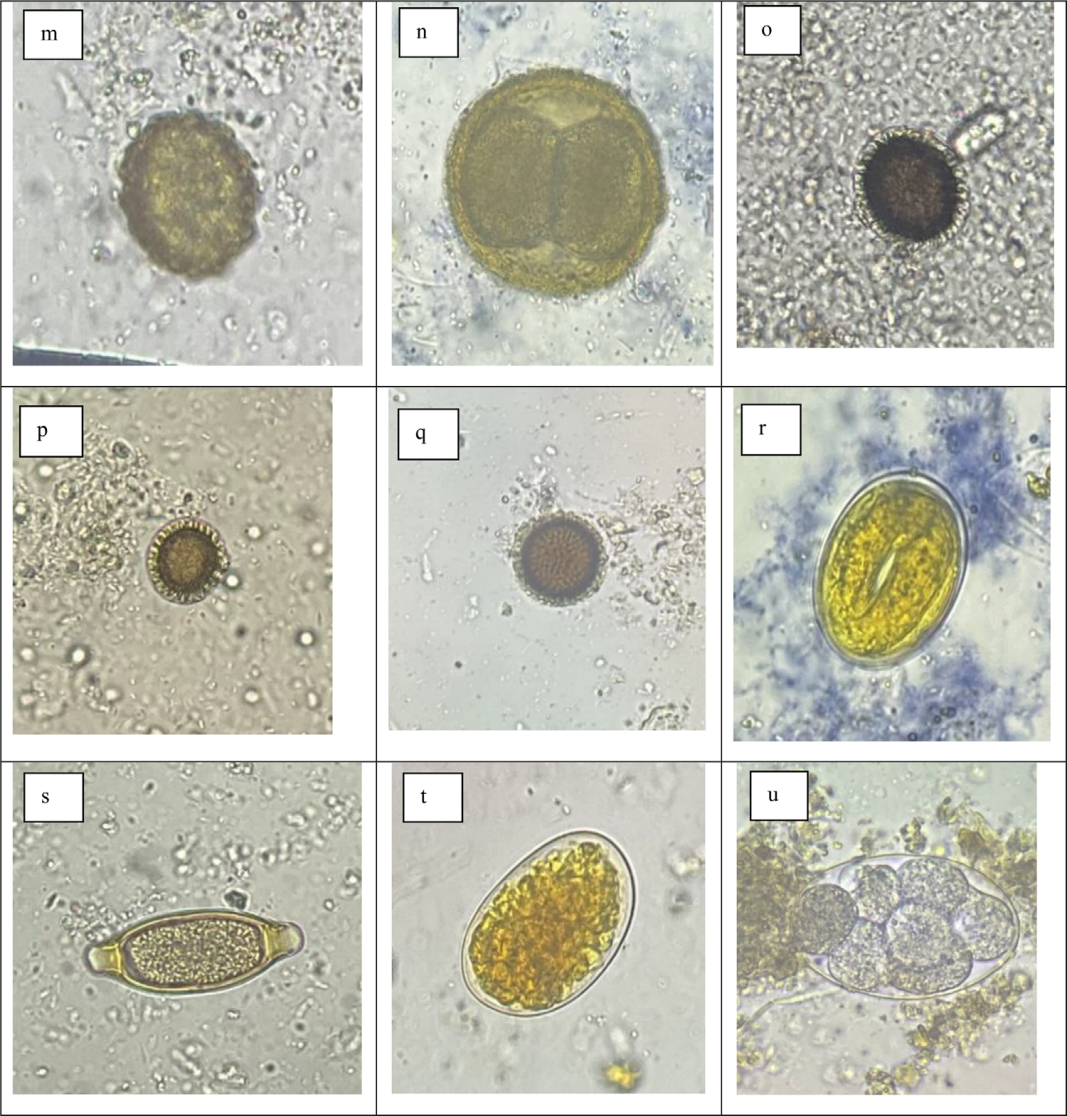

### 3.2 Intensity of IP Species in Humans and Domestic Animals

The intensity of various IP species was studied, and it was found that light infection (+) was highest compared to moderate (++) and heavy infections (+++). In contrast, pigs had a moderate intensity of *Entamoeba* spp. and *Eimeria* spp. The dog hookworm also had a similar moderate intensity. Notably, no cases of heavy infection were observed in dogs and cattle. Significant differences were recorded regarding the light infection and heavy infection on humans, light infection on cattle and heavy infection on pigs (*p* < 0.05) (Supporting Information 1).

### 3.3 Concurrency Pattern of All and Zoonotic Parasites in all Hosts

**FIGURE 3:**
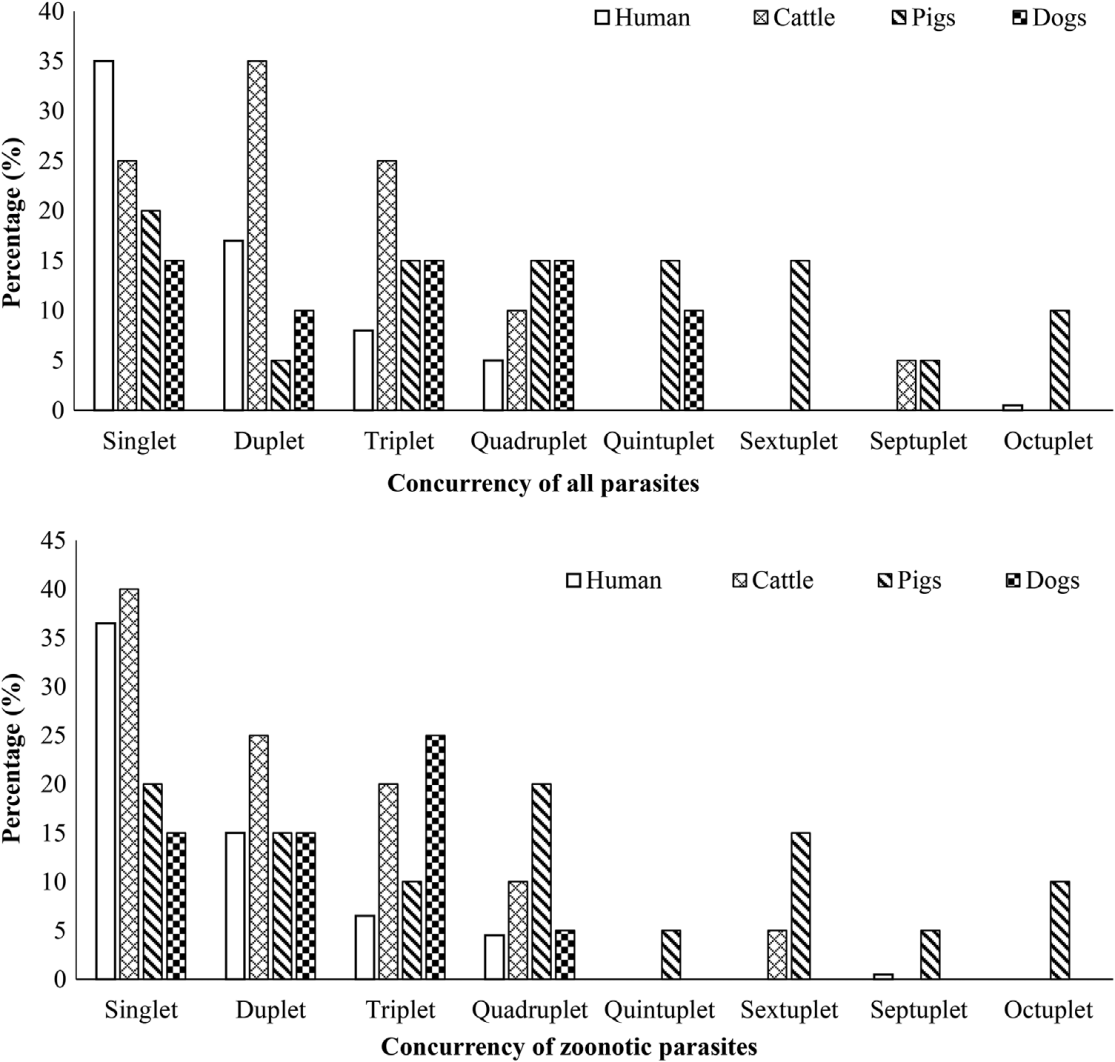
Concurrency of IP species. (a) Concurrency of all parasites in different hosts. (b) Concurrency of zoonotic parasites in different hosts.

Humans showed a predominant mono‐parasitism over poly‐parasitism (35% vs. 30.5%). Conversely, their domestic animals exhibited a greater occurrence of poly‐parasitism compared to mono‐parasitism (cattle, 75% vs. 25%; pigs, 80% vs. 20% and dogs, 50% vs. 15%) (Figure 3a). Similarly, the concurrency of zoonotic parasites also revealed similar results; single infection in humans (36.5%) and multiple infection in all domestic animals, that is, cattle (60%), pigs (80%) and dogs (45%), to be more common (Figure 3b).

### 3.4 Risk Factors in Humans

Significant associations were observed between specific IP species and participants’ characteristics. For example, association of *Cryptosporidium* spp. and *Ascaris* spp. with occupation type and feeding habit (*p* < 0.05), *Giardia* with education status (*p* < 0.05), Acanthocephala sp. with symptoms (*p* < 0.05), *E. coli* with drug intake history (*p* < 0.05), *Endolimax nana* and Taeniid with checkup priority (*p* < 0.05) and *Strongyloides* and *E. nana* with checkup priority (*p* < 0.05). However, risk factor (feeding habit) analysis using RR, OR, and LR indicated poor strength of association with particular IP species (Table 2).

**TABLE 2:** Risk factors of IP species in humans.

Variables	Criteria	*Entamoeba coli*	*Entamoeba histolytica* *	*Endolimax nana* *	*Iodamoeba buetschlii* *	*Blastocystis hominis* *	*Giardia* *	*Balantidium coli* *	*Cryptosporidium* spp.*	*Cyclospora* spp.*	Taeniid*	*Ascaris* spp.*	*Strongyloides* sp.*	Hookworm*	Strongyle*	Acanthocephala sp.*
Sex	Male (*n* = 97)	12	6	6	1	3	1	6	17	11	13	15	10	2	2	1
	Female (*n* = 103)	18	12	3	6	3	4	2	19	7	24	17	8	2	1	0
*p*‐values		ns	ns	ns	ns	ns	ns	ns	ns	ns	ns	ns	ns	ns	ns	ns
Age groups	18–40 (*n* = 89)	16	7	7	5	3	4	1	17	8	14	15	5	3	2	0
	41–60 (*n* = 69)	7	7	1	2	2	1	5	12	6	12	11	8	0	0	0
	Above 60 (*n* = 42)	7	4	1	0	1	0	2	7	4	11	6	5	1	1	1
		ns	ns	ns	ns	ns	ns	ns	ns	ns	ns	ns	ns	ns	ns	ns
Education status	None (*n* = 79)	15	8	2	2	3	0	4	17	7	17	13	10	1	3	0
	Up to eighth grade (*n* = 72)	9	5	2	3	1	2	3	13	6	14	9	5	2	0	0
	9th–10th grade (*n* = 20)	3	3	2	0	1	0	0	2	2	1	3	2	1	0	0
	≥ 11th grade (*n* = 29)	3	2	3	2	1	3	1	4	3	5	7	1	0	0	1
*p*‐values		ns	ns	ns	ns	ns	*P* < 0.05	ns	ns	ns	ns	ns	ns	ns	ns	ns
Occupation type	Unemployed (*n* = 12)	2	3	3	0	0	1	0	1	0	1	1	0	0	0	1
	Student (*n* = 1)	0	0	0	0	0	0	0	0	0	0	0	0	0	0	0
	Farmer (*n* = 156)	25	13	4	6	5	3	5	27	14	32	22	14	4	2	0
	Businessman (*n* = 4)	0	0	0	0	0	0	1	0	0	1	1	0	0	0	0
	Teacher (*n* = 13)	1	1	1	0	0	1	0	2	2	1	4	0	0	0	0
	Labourer (*n* = 8)	2	0	0	1	1	0	2	3	1	2	2	2	0	0	0
	Government job holder (*n* = 3)	0	1	1	0	0	0	0	2	0	0	1	1	0	0	0
	Trekking guide (*n* = 1)	0	0	0	0	0	0	0	0	0	0	1	1	0	0	0
	Carpenter (*n* = 2)	0	0	0	0	0	0	0	1	1	0	0	0	0	1	0
*p*‐values		ns	ns	ns	ns	ns	ns	ns	*p* < 0.05	ns	ns	p<0.05	ns	ns	ns	ns
Household size	Less than 5 (*n* = 129)	21	13	4	3	4	3	6	25	11	21	22	11	2	2	1
	Equal to or more than 5 (*n* = 71)	9	5	5	4	2	2	2	11	7	16	10	7	2	1	0
*p‐*values		ns	ns	ns	ns	ns	ns	ns	ns	ns	ns	ns	ns	ns	ns	ns
Source of drinking water	Tap water (*n* = 200)	30	18	9	7	6	5	8	36	18	37	32	18	4	3	1
	Well water (*n* = 0)	0	0	0	0	0	0	0	0	0	0	0	0	0	0	0
*p*‐values		ns	ns	ns	ns	ns	ns	ns	ns	ns	ns	ns	ns	ns	ns	ns
Consumption habit of drinking water	Without chemical treatment (*n* = 192)	30	18	9	7	6	5	8	34	17	36	30	18	4	3	1
	After boiling (*n* = 8)	0	0	0	0	0	0	0	2	1	1	2	0	0	0	0
*p*‐values		ns	ns	ns	ns	ns	ns	ns	ns	ns	ns	ns	ns	ns	ns	ns
Hand wash before meals and after defecation	With water only (*n* = 13)	1	0	2	0	0	0	1	1	1	4	5	1	0	0	0
	With water + soil (*n* = 4)	2	0	0	1	1	0	0	1	1	0	0	0	0	0	0
	With water + ash (*n* = 6)	1	1	0	0	0	0	0	2	0	0	1	0	1	0	0
	With water + soap (*n* = 177)	26	17	7	6	5	5	7	32	16	33	26	17	3	3	1
*p*‐values		ns	ns	ns	ns	ns	ns	ns	ns	ns	ns	ns	ns	ns	ns	ns
Type of latrine	Permanent latrine (*n* = 198)	30	18	9	7	6	5	8	36	18	36	31	18	4	3	1
	Temporary Latrine (*n* = 2)	0	0	0	0	0	0	0	0	0	1	1	0	0	0	0
*p*‐value		ns	ns	ns	ns	ns	ns	ns	ns	ns	ns	ns	ns	ns	ns	ns
Use of sandals and shoes	Always (*n* = 183)	26	18	9	6	5	5	7	34	17	33	29	16	3	3	1
	Sometimes (*n* = 17)	4	0	0	1	1	0	1	2	1	4	3	2	1	0	0
*p*‐values		ns	ns	ns	ns	ns	ns	ns	ns	ns	ns	ns	ns	ns	ns	ns
Feeding habit	Non‐vegetarian (*n* = 198)	30	17	9	7	6	5	8	34	17	36	30	18	4	3	1
	Vegetarian (*n* = 2)	0	1	0	0	0	0	0	2	1	1	2	0	0	0	0
*p*‐values RR (95% CI), OR (85% CI), and LR		ns	ns	ns	ns	ns	ns	ns	*P *< 0.05 0.1717 (0.1265–0.2332) 0.04195 (0.001968–0.893) 0.9444	ns	ns	*p *< 0.05 0.1515 (0.1090–0.2107) 0.03620 (0.001695–0.773) 0.9375	ns	ns	ns	ns
Pork consumption	Yes (*n* = 194)	30	16	8	7	6	4	8	35	18	35	32	18	4	3	1
	No (*n* = 6)	0	2	1	0	0	1	0	1	0	2	0	0	0	0	0
*p*‐values		ns	ns	ns	ns	ns	ns	ns	ns	ns	ns	ns	ns	ns	ns	ns
Pigs nearby	Yes (*n* = 152)	21	14	7	6	5	4	6	28	15	32	25	13	3	3	1
	No (*n* = 48)	7	4	2	1	1	1	2	8	3	5	7	5	1	0	0
*p*‐values		ns	ns	ns	ns	ns	ns	ns	ns	ns	ns	ns	ns	ns	ns	ns
Knowledge of IPIs	Yes (*n* = 143)	17	14	7	4	2	4	6	27	13	23	19	12	2	2	1
	No (*n* = 57)	13	4	2	3	4	1	2	9	5	14	13	6	2	1	0
*p*‐values		ns	ns	ns	ns	ns	ns	ns	ns	ns	ns	ns	ns	ns	ns	ns
Symptoms	Diarrhoea (*n* = 12)	1	0	3	0	2	1	0	4	1	1	1	1	0	1	1
	Constipation (*n* = 1)	0	0	0	0	0	0	0	0	1	0	0	0	0	0	0
	Dysentery (*n* = 0)	0	0	0	0	0	0	0	0	0	0	0	0	0	0	0
	Vomiting (*n* = 6)	0	1	0	0	1	0	0	1	0	2	3	1	1	0	0
	Stomachache (*n* = 28)	6	2	1	0	0	1	1	4	2	6	6	2	0	0	0
	Other (extra‐intestinal symptoms) (*n* = 1)	0	0	0	0	0	0	0	0	0	1	0	0	0	0	0
	Diarrhoea + constipation (*n* = 8)	0	0	0	0	0	0	0	0	1	1	3	1	0	0	0
	Diarrhoea + vomiting (*n* = 3)	0	0	0	0	0	0	0	0	0	0	0	1	0	0	0
	Diarrhoea + stomach‐ache (*n* = 12)	3	0	0	0	0	0	4	4	0	2	4	2	0	0	0
	Dysentery + stomach‐ache (*n* = 5)	1	1	0	0	0	0	0	2	0	0	1	0	0	0	0
	Vomiting + stomach‐ache (*n* = 7)	2	1	1	1	1	0	0	3	2	4	0	0	1	1	0
	Equal to or more than three symptoms (*n* = 31)	3	2	1	1	0	1	0	4	4	5	4	2	2	0	0
	None (*n* = 84)	14	10	3	5	2	2	3	14	7	15	10	8	0	1	0
	All (*n* = 2)	0	1	0	0	0	0	0	0	0	0	0	0	0	0	0
*p*‐values		ns	ns	ns	ns	ns	ns	ns	ns	ns	ns	ns	ns	ns	ns	*p* < 0.05
Checkup priority	Hospital (*n* = 10)	1	0	0	0	0	1	0	0	2	6	3	2	0	0	0
	Medical (*n* = 2)	0	0	0	0	0	0	1	0	1	0	1	0	0	0	0
	Health post (*n* = 119)	19	10	4	6	4	2	4	20	10	20	19	10	2	3	1
	Dhami Jhakri (*n* = 37)	6	5	1	0	1	1	3	9	1	8	5	3	0	0	0
	Jadibuti and Ayurveda (*n* = 2)	1	0	0	0	0	0	0	0	0	0	1	0	0	0	0
	Nowhere (*n* = 30)	3	3	4	1	1	1	0	7	4	3	3	3	2	0	0
*p*‐values		ns	ns	*p* < 0.05	ns	ns	ns	ns	ns	ns	*p* < 0.05	ns	ns	ns	ns	ns
Intake of drugs	Before 1 month (*n* = 3)	0	0	0	0	0	0	0	0	0	2	1	1	0	0	0
	2–3 months (*n* = 13)	0	1	0	0	0	0	0	3	0	1	3	0	0	0	0
	4–6 months (*n* = 16)	0	1	0	1	0	0	1	3	1	2	1	1	1	0	0
	7–12months (*n* = 12)	0	1	0	1	0	0	0	0	0	2	0	0	0	1	0
	Before 12 months (*n* = 58)	6	5	2	0	2	2	2	11	6	6	9	5	0	0	1
	No (*n* = 98)	24	10	7	5	4	3	5	19	11	24	18	11	3	2	0
*p*‐values		p<0.05	ns	ns	ns	ns	ns	ns	ns	ns	ns	ns	ns	ns	ns	ns
Stool colour and consistency	Yellow/solid (*n* = 34)	2	3	0	0	0	0	0	9	2	5	4	0	0	1	0
	Yellow/soft (*n* = 46)	7	2	1	3	2	0	4	8	6	6	8	3	0	1	1
	Yellow/diarrhoea (*n* = 6)	0	0	0	0	0	1	0	0	0	1	2	2	1	0	0
	Brown/solid (*n* = 66)	13	8	4	2	1	4	2	12	4	18	15	8	2	0	0
	Brown/soft (*n* = 44)	8	5	4	1	3	0	2	7	5	6	3	4	1	1	0
	Brown/diarrhoea (*n* = 3)	0	0	0	0	0	0	0	0	1	1	0	1	0	0	0
	Green/solid (*n* = 1)	0	0	0	0	0	0	0	0	0	0	0	0	0	0	0
*p*‐values		ns	ns	*p* < 0.05	ns	ns	ns	ns	ns	ns	ns	ns	*p* < 0.05	ns	ns	ns

^*^ denotes the zoonotic parasites.

### 3.5 Common IP Species in Humans and Domestic Animals

**FIGURE 4:**
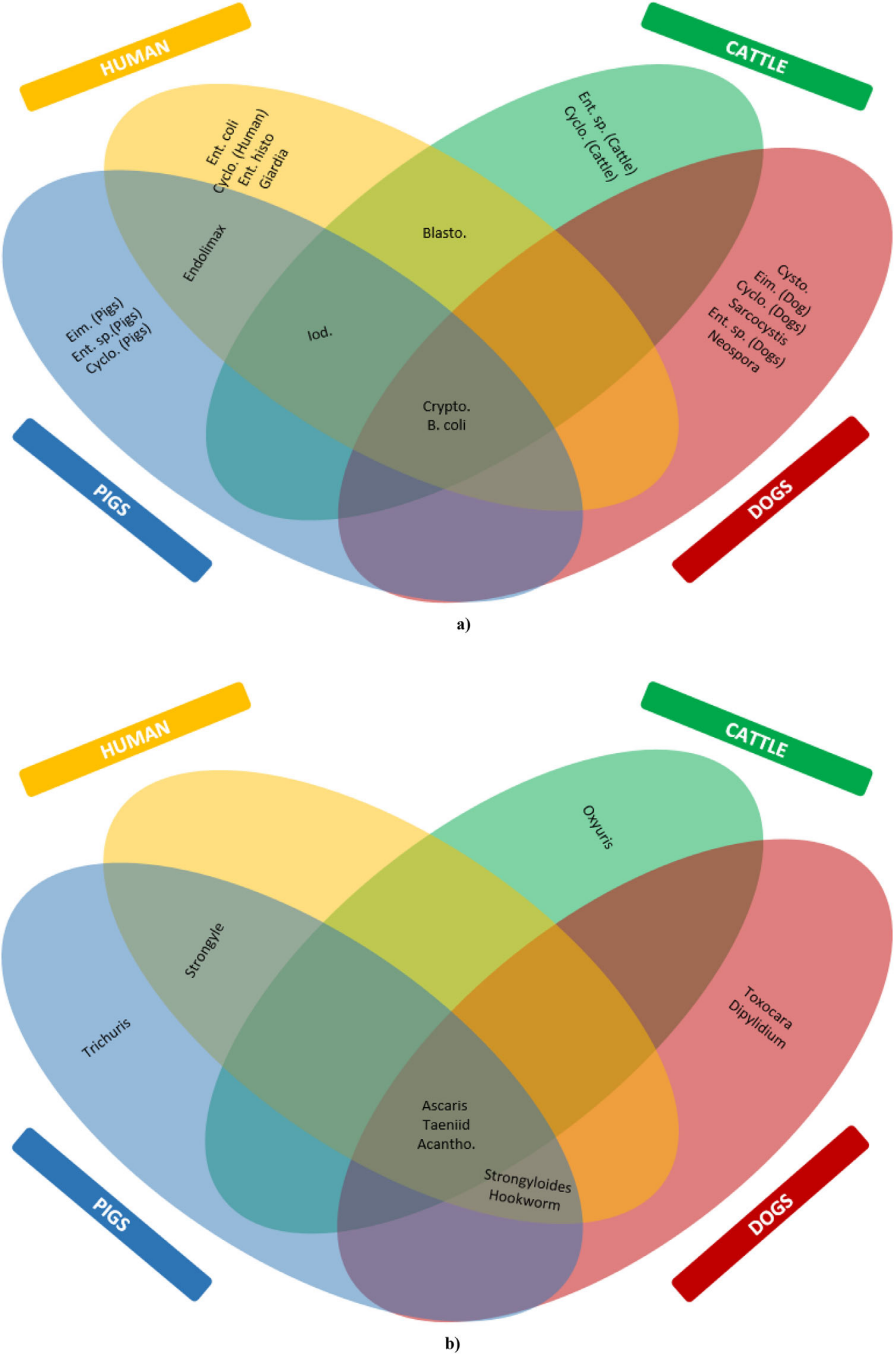
Common IP species in humans and domestic animals. (a) Protozoan species, (b) Helminth species.

A comparative assessment revealed some zoonotic IP species like *Cryptosporidium* spp., *B. coli*, *Ascaris* spp., taeniid and Acanthocephala spp. in both humans and domestic animals (Figure 4).

### 3.6 Shared Parasite Species Between Humans and Domestic Animals in the Same Household

We analysed the shared parasites between humans and different hosts sampled within the same household. In the comparative analysis of 40 faecal samples collected from pigs and humans living in the same households (20 samples each), several zoonotic parasites were found to be shared between these two hosts. Notably, *Cryptosporidium* spp. were principally detected in four pigs and four human samples in the same households, indicating a potential risk of cross‐species transmission, which highlights the overlap in parasitic infections between two hosts in a close contact setting. Similarly, taeniid species were found to be shared between cattle and humans, as well as between dogs and humans residing in the same households. Additionally, *Entamoeba* spp. were also mainly found in two cattle and two humans in the same household (Table 3).

**TABLE 3:** Shared IP species found between pigs and humans, or cattle and humans or dogs and humans. Numbers of parasite‐positive samples were assessed by the number of faecal samples of domestic animals and humans that reared them.

Shared parasites	Pigs + humans (*N* = 20 + 20 → 40)	Cattle + humans (*N* = 20 + 20 → 40)	Dogs + humans (*N* = 20 + 20 → 40)
*Entamoeba* spp.*	0	2	0
Balantidium coli*	2	1	0
*Cryptosporidium* spp.*	4	0	1
Taeniid*	1	2	2
*Ascaris* spp.*	1	1	1
Hookworm*	1	0	0
*Strongyloides* sp.*	1	0	0
Strongyle*	1	0	0
Endolimax spp.*	1	0	0
Total	12	6	4
Shared number of species	8	4	3

^*^ denotes the zoonotic parasites.

### 3.7 Parasite Load in Domestic Animals

*Cryptosporidium* spp. were concomitantly found with other parasites of zoonotic importance; however, they were mainly seen with *Eimeria* spp. in three pigs (75%) (Supporting Information 2a). Those cattle that had Taeniid species had different parasitic species of zoonotic significance (Supporting Information 2b). Dogs that were positive for Taeniid species were also positive for various zoonotic protozoans and helminths (Supporting Information 2c).

## 4 Discussion

An overall prevalence of 70.77% was reported for IPIs among humans and domestic animals. Specifically, the prevalence rate in humans was 65.5% indicating a significant burden of IPIs in MDRM, Solukhumbu, Nepal. The prevalence rate of our study was higher than the findings from other regions of Nepal (1.41%–42.46%, *N* = 71–205), such as 1.41% (1/71) in Dolakha district (Dhakal et al. 2024), 22% (44/200) in Arghakhanchi district (Gautam et al. 2024), and 42.46% (76/179) in Bara district (Chaudhary and Ghimire 2025). Other studies, however, recorded a higher prevalence than our current findings from different indigenous communities of Nepal, such as Musahar (81%, 162/200) (Yadav et al. 2024) and Chepangs (100%, 100/100) (Adhikari et al. 2021b). When compared to the global context, the prevalence rate observed in our study was considerably higher than those reported in various countries, such as Southeast China (1.80%, *N* = 23,552) (Feng et al. 2021), Slovakia (5.95%, *N* = 2503) (Ihnacik et al. 2025), Mongolia (6.4%, *N* = 419) (Barnes et al. 2021), Pakistan (21%, *N* = 204) (Karim et al. 2024) and Argentina (30.6%, *N* = 96) (Scavuzzo et al. 2023). Previous studies on humans recorded four species (Scavuzzo et al. 2023), five species (Gautam et al. 2024; Karim et al. 2024), nine species (Yadav et al. 2024; Chaudhary and Ghimire 2025), 10 species (Sebaa et al. 2021) and 14 species (Adhikari et al. 2021b).

Likewise, this study revealed the prevalence of IP species in three domestic animals, with rates of 65% in dogs and 100% in cattle and pigs. In case of cattle, studies in Nepal recorded prevalence rates of 51.32%–72% (Thapa et al. 2022; Bastakoti et al. 2023; Patel et al. 2025), while studies in Ethiopia and Thailand reported 67.2% and 96.09%, respectively (Thanasuwan et al. 2021; Tiele et al. 2023). The prevalence rate of IP species among dogs was reported to be 58% in Morocco (Idrissi et al. 2022), 59.5% in Suryabinayak, Nepal (Sukupayo and Tamang 2023), 62% in Bangladesh (Nath et al. 2022) and 66.67% in domestic dogs in Humla, Nepal (Acharya et al. 2025). Regarding IP species of pigs, studies in Nepal documented the prevalence rates of 86.5%–91% (Adhikari et al. 2021a; Chaudhary et al. 2023), while global studies, like Bangladesh reported 79% (Nath et al. 2022), Thailand reported 85.19% (Thanasuwan et al. 2024) and Argentina reported 90.4% (Alegre et al. 2024).

Regarding the diversity of IP species in the people of MDRM, we recorded a total of 15 different species of parasites. Compared to previous national and global studies, our study revealed a high diversity of IP species, in which *Cryptosporidium* spp. and taeniid were the most encountered IP species in humans and dogs. Similarly, *Entamoeba* spp. and Ascarid in cattle and *B. coli* and Strongyle in pigs were the most commonly observed parasites. Compared to current numbers, 15 species in dogs, 10 species in cattle and 14 species in pigs, eight species in Morocco (Idrissi et al. 2022), 19 species in Humla, Nepal (Acharya et al. 2025) and 23 species in Lalitpur, Nepal (Adhikari et al. 2023) from dogs; six species (Thanasuwan et al. 2021; Patel et al. 2025) from cattle and 12 species (Chaudhary et al. 2023) and 14 species (Adhikari et al. 2021a) from pigs were recorded.

The presence of diverse genera of parasites in our study area shows the greater diversity and abundance of IP species. These variations may be attributed to several factors, such as sociodemographic characteristics, behavioural practices, animal husbandry practices, deworming schedules and environmental aspects like geographical location, altitude and climatic conditions. Additionally, differences in study design, sample size and laboratory techniques employed for sample processing might also have contributed to discrepancies in prevalence rates (Adhikari et al.; Adhikari et al. 2021a; Adhikari et al. 2023; Chaudhary and Ghimire 2025).

It is widely accepted that several risk factors of IPIs exist in many regions of the world, including Nepal. In this study, occupation type and feeding habit, symptoms, checkup priorities, intake of drugs and stool colour and consistency were associated with one or more IPIs. For example, feeding habit is a risk factor for IPIs in previous studies (Gautam et al. 2024; Chaudhary and Ghimire 2025). Meat consumption enhances the distribution of the larval forms, eggs and adults of the IP species. For example, pork consumption is strongly associated with a high chance of *Taenia* infection around the globe, as inadequately cooked pork may contain larval forms of *Taenia* species (WHO  2022). Although the current data were statistically significant based on chi‐square analysis, the OR, RR and LR analyses did not generate significance, particularly due to unequal sampling sizes. There were only two vegetarians and 198 non‐vegetarians, and their respective prevalence rates of 100% and 17.17% indicated that non‐vegetarians had a statistically significant 82.83% lower risk of parasitosis than vegetarians, producing an estimate potentially unstable, prone to sampling error, and clinically irrelevant OR, RR and LR without any significance. Similar results have been obtained for *Ascaris* spp. during risk analysis, suggesting this result should be interpreted with caution and warrants confirmation in larger cohorts.

Similarly, lack of timely intake of drugs is a critical factor in IPIs. In Nepal, drug distribution is usual. However, deworming practices are not completely established, especially in rural areas where people usually do not go to the hospital or the nearby health offices to take drugs. Limited drug availability in rural health facilities may have influenced the infection patterns observed in our study. For instance, in the MDRM health post of our study area, only the anthelmintic Albendazole was available, while no provision of anti‐protozoal drugs was observed (T.K. Tamang, Focal Group Discussion, Personal Communication, 2024). Most participants (*n* = 119) reported visiting the health post for any abdominal discomfort or other IPIs‐related symptoms. However, the lack of access to anti‐protozoal treatment could explain the higher prevalence of protozoan infections in the study population, as untreated cases might have persisted and contributed to ongoing transmission. Although half of the population took Albendazole within 1–6 months, they were still positive for helminths, indicating the possibility of resistance developed by the bodies of the local people, similar to problems addressed by previous literature that highlighted the challenges of anthelmintic resistance (Geerts and Gryseels 2001; Nixon et al. 2020; Harshita and Nonika 2024). The intestinal helminth species like *Necator americanus* resistance to mebendazole (Mali), *Ancylostoma duodenale* resistance to pyrantel (Australia), *Schistosoma mansoni* resistance to oxamniquine (Brazil) and praziquantel (Senegal and Egypt) and *Onchocerca volvulus* resistance to ivermectin (West Africa) are some of the reported cases of anti‐helminthic resistance in humans (Geerts and Gryseels 2001). In the context of cryptosporidiosis, most (30%) people were suffering from diarrhoea, stomach‐ache and vomiting. This coccidian infection is caused by *Cryptosporidium*, which could be cured by TMP‐SMZ, that is, Bactrim (Ghimire et al. 2010); however, in the study area, there was no proof of routine examination of *Cryptosporidium* oocysts and subsequent treatment as the health workers and laboratory professionals usually skip recommending and detecting these coccidia. Importantly, education status has been a risk factor for Giardiasis. *Giardia*, a waterborne protozoan parasite, is usually transmitted via consumption of contaminated water and easily spreads among people, who usually practice drinking water without treatment if not properly guided (Nygård et al. 2006).

In this microscopic study, none of the hosts had trematode parasites similar to previous research works (Sukupayo and Tamang 2023; Dhakal et al. 2024; Chaudhary and Ghimire 2025). This might be because of the absence of local ponds or similar water bodies that serve as habitats for the snails and other intermediate hosts required to complete the lifecycle of trematodes. Moreover, agricultural practices such as terrace farming, rotational livestock grazing and keeping dogs as safeguards restrict the free‐ranging domestic animals. It results in the breakdown of the trematode lifecycle, even if it may be present in the snail‐containing vegetations or water.

The study recorded 19 species of zoonotic parasites, in which *Cryptosporidium* spp., *B. coli*, *Ascaris* spp., taeniid and Acanthocephala were shared by all hosts. *Cryptosporidium* spp. has been reported in many hosts, like dogs (Adhikari et al. 2023), pigs (Adhikari et al. 2021a) and cattle (Buchanan et al. 2025), which are transmitted zoonotically as well as clinically through close contact with companion animals like our current non‐human hosts. The latter four shared IP species are usually transmitted between humans and pigs (Agustina et al. 2023) or cattle (Shams et al. 2021) or dogs (Alegría‐Morán et al. 2021; Otranto et al. 2021). These parasites are also transmitted directly between humans and the animal hosts through faecal‐borne, soil‐borne, finger‐borne and vector (mechanical)‐borne modes. The spreading condition is furnished chiefly in the agricultural landscapes where humans and other hosts coexist, and such proximity is found in the current study area. It is evident by the detection of common IP species in the faecal samples of humans and animals living in the same households, raising the possibility of complex zoonosis in this rural setting. Further one health approach of understanding molecular mechanisms of existing parasitic zoonosis and reducing and preventing it by integrated treatment and awareness interventions.

This study has a few limitations. First, this is a descriptive study. The absence of a case‐control study limits the availability of data from one point to reflect the parasitic infection levels existing in the study sites. Second, the laboratory diagnosis technique relied solely on morphology, as no molecular diagnostic tools, like polymerase chain reaction, were used. The detection of parasites through morphology has limitations in sensitivity and specificity. Also, a relatively smaller sample size of both human and domestic animals may have amplified type I and type II errors. Despite these limitations, we have maintained our study, both qualitatively and quantitatively strong. For example, a thorough sampling, support of focal group discussion during questionnaire and field survey, and repetitive examinations of laboratory stool samples have been scientifically implemented to nullify the false positive or false negative results.

## 5 Conclusion

In conclusion, this study documented that more than half of the human and domestic animal population have IPIs in the rural setting of Solukhumbu, Nepal. These findings reinforce the need to consider deworming campaigns with community health education as a preventive measure for both humans and domestic animals. The study also highlights that the IP species are shared among humans and nearby domestic animals probably through different routes, such as via contact with contaminated soil, food, water, and faeces. Therefore, further One Health studies on human and environmental samples should be carried out to identify the epidemiologic routes of zoonoses among different hosts. These interventions will lower the associated parasitic disease risks by improving the knowledge, attitude, and practices in the context of the rural Nepalese community.

## Author Contributions

**Prince Rai**: conceptualization, methodology, manuscript writing, final manuscript preparation. **Tirth Raj Ghimire**: conceptualization, methodology, manuscript writing, final manuscript preparation, supervision

## Ethics Statement

We are thankful to Ward Number 5 Office, MDRM, Solukhumbu, Nepal (Dispatch Number: *209*) for granting permission to work in the study area. We acknowledge the Institutional Research Committee, Institute of Science and Technology, Tribhuvan University, for their ethical approval to conduct the research (Ref. Number: 167/080/081).

## Conflicts of Interest

The authors declare no conflicts of interest.

## Funding

The authors have nothing to report.

## Disclosure

The lead author, Prince Rai, affirms that this manuscript is an honest, accurate, and transparent account of the study being reported; that no important aspects of the study have been omitted and that any discrepancies from the study as planned (and, if relevant, registered) have been explained.

## Supporting information



Supplementary 1. Intensity of IP species in humans and domestic animals.
Supplementary 2a. Presence of different species of parasites with shared parasites of pigs and humans.
Supplementary 2b. Presence of different species of parasites with shared parasites of cattle and humans.
Supplementary 2c. Presence of different species of parasites, including shared parasites of dogs and humans

## Data Availability

The authors confirm that all data supporting the study's findings are available in the Data S1 of this article.
