# Supplementary material for: Intestinal Parasites of Zoonotic Significance in Human and Domestic Animals in a Rural Setting in Nepal
Source: Vet Med Sci. 2025 Dec 11;12(1):e70728. doi: 10.1002/vms3.70728 (PMC12696394; doi:10.1002/vms3.70728)
Supplement: Supplementary file 1 — Supplementary 1. Intensity of IP species in humans and domestic animals. Supplementary 2a. Presence of different species of parasites with shared parasites of pigs and humans. Supplementary 2b. Presence of different species of parasites with shared parasites of cattle and humans. Supplementary 2c. Presence of different species of parasites, including shared parasites of dogs and humans [file VMS3-12-e70728-s001.docx]

**Supplementary 1.** Intensity of IP species in humans and domestic animals

| **Type of hosts** | **Humans (n=200)** | | | **Cattle (n=20)** | | | **Pigs (n=20)** | | | **Dogs (n=20)** | | |
| --- | --- | --- | --- | --- | --- | --- | --- | --- | --- | --- | --- | --- |
| **Intensity Level** | **+** | **++** | **+++** | **+** | **++** | **+++** | **+** | **++** | **+++** | **+** | **++** | **+++** |
| **Sarcodina** | | | | | | | | | | | | |
| *Entamoeba* spp. * | 25 | 1 | 4 | 16 | 1 | -- | 1 | 6 | 4 | 1 | -- | -- |
| *Entamoeba histolytica* * | 15 | 1 | 2 | -- | -- | -- | -- | -- | -- | -- | -- | -- |
| *Endolimax* spp. * | 9 | -- | -- | -- | -- | -- | 2 | -- | -- | -- | -- | -- |
| *Iodamoeba* spp. * | 6 | 1 | -- | 1 | -- | -- | 1 | -- | -- | -- | -- | -- |
| *Blastocystis* spp. * | 6 | -- | -- | 2 | -- | -- | -- | -- | -- | -- | -- | -- |
| **Flagellata** | | | | | | | | | | | | |
| *Giardia* * | 3 | 2 | -- | -- | -- | -- | -- | -- | -- | -- | -- | -- |
| **Ciliata** | | | | | | | | | | | | |
| *Balantidium coli* * | 8 | -- | -- | 4 | -- | -- | 10 | 1 | -- | 1 | -- | -- |
| **Apicomplexa** | | | | | | | | | | | | |
| *Cryptosporidium* spp. * | 30 | 5 | 1 | 2 | 2 | -- | 3 | 1 | 1 | 5 | 1 | -- |
| *Cyclospora* spp. * | 16 | 2 | -- | 5 | -- | -- | 1 | -- | -- | 1 | -- | -- |
| *Eimeria* spp. | -- | -- | -- | -- | -- | -- | 3 | 4 | 1 | 2 | 1 | -- |
| *Cystoisospora* sp. | -- | -- | -- | -- | -- | -- | -- | -- | -- | 3 | 2 | -- |
| *Sarcocystis* sp. * | -- | -- | -- | -- | -- | -- | -- | -- | -- | 1 | -- | -- |
| *Neospora caninum* | -- | -- | -- | -- | -- | -- | -- | -- | -- | 2 | -- | -- |
| **Cestoda** | | | | | | | | | | | | |
| Taeniid * | 35 | 2 | -- | 7 | -- | -- | 3 | 1 | 2 | 5 | -- | -- |
| *Dipylidium caninum* * | -- | -- | -- | -- | -- | -- | -- | -- | -- | 1 | -- | -- |
| **Nematoda** | | | | | | | | | | | | |
| *Ascaris* spp. * | 33 | 1 | -- | 9 | -- | -- | 4 | 2 | -- | 3 | -- | -- |
| *Toxocara canis* * | -- | -- | -- | -- | -- | -- | -- | -- | -- | 2 | 1 | -- |
| *Strongyloides* spp. * | 15 | 3 | -- | -- | -- | -- | 3 | 2 | -- | 1 | -- | -- |
| Hookworm * | 2 | 2 | -- | -- | -- | -- | 2 | 2 | -- | 1 | 3 | -- |
| Strongyle * | 3 | -- | -- | -- | -- | -- | 9 | 5 | -- | -- | -- | -- |
| *Oxyuris* sp. | -- | -- | -- | 1 | -- | -- | -- | -- | -- | -- | -- | -- |
| *Trichuris* sp. * | -- | -- | -- | -- | -- | -- | 2 | -- | -- | -- | -- | -- |
| **Acanthocephala** | | | | | | | | | | | | |
| Acanthocephala spp. * | 1 | -- | -- | 1 | -- | -- | 1 | -- | -- | 1 | -- | -- |
| **p-value** | p<0.05 | ns | p<0.05 | p<0.05 | ns | -- | ns | ns | p<0.05 | ns | ns | -- |
| **Total Number** | **207** | **20** | **7** | **48** | **3** | **0** | **45** | **24** | **8** | **30** | **8** | **0** |

***Note – Symbol (--) indicates absent, ns- not significant***

**Supplementary 2**

**Supplementary 2a:** Presence of different species of parasites with shared parasites of pigs and humans

| **Shared parasites** | **Pigs (P_1_)** | **Pigs (P_2_)** | **Pigs (P_3_)** | **Pigs (P_4_)** |
| --- | --- | --- | --- | --- |
| *Balantidium coli* * A | *Eimeria* spp.  Strongyle  *Ascaris* spp.  Taeniid |  |  |  |
| *Balantidium coli* * B |  | **None** |  |  |
| *Cryptosporidium* sp. * A | *Eimeria* spp.  *Balantidium coli*  *Entamoeba* spp.  Strongyle  Acanthocephala sp. |  |  |  |
| *Cryptosporidium* sp. * B |  | *Eimeria* spp.  Strongyle  *Ascaris* spp.  Taeniid |  |  |
| *Cryptosporidium* sp. * C |  |  | Strongyle  *Ascaris* spp.  *Strongyloides* sp. |  |
| *Cryptosporidium* sp. * D |  |  |  | *Entamoeba* spp.  *Eimeria* spp.  *Endolimax nana Balantidium coli*  Strongyle  *Strongyloides* sp. |
| Taeniid * A | *Entamoeba* spp.  Strongyle  Acanthocephala sp. |  |  |  |
| *Ascaris* spp. * A | *Balantidium coli*  *Eimeria* spp.  *Entamoeba* spp.  *Strongyloides* sp.  Strongyle |  |  |  |
| Hookworm * A | *Eimeria* spp.  Strongyle |  |  |  |
| *Strongyloides* sp. * A | *Eimeria* spp.  *Entamoeba* spp.  *Endolimax nana Cryptosporidium* sp  *Balantidium coli*  Strongyle |  |  |  |
| Strongyle * A | *Entamoeba* spp.  Taeniid  Strongyle  Acanthocephala sp. |  |  |  |

**Supplementary 2b:** Presence of different species of parasites with shared parasites of cattle and humans

| **Shared parasites** | **Cattle (C_1_)** | **Cattle (C_2_)** |
| --- | --- | --- |
| *Entamoeba* spp. A | None |  |
| *Entamoeba* spp. B |  | Taeniid |
| *Balantidium coli* * A | *Entamoeba* spp. |  |
| Taeniid * A | *Blastocystis hominis*  *Ascaris* spp. |  |
| Taeniid * B |  | *Entamoeba* spp.  *Cryptosporidium* sp. |
| *Ascaris* spp. * A | *Balantidium coli*  *Ascaris* spp.  Taeniid |  |

**Supplementary 2c:** Presence of different species of parasites, including shared parasites of dogs and humans

| **Shared parasites** | **Dogs (D_1_)** | **Dogs (D_2_)** |
| --- | --- | --- |
| *Cryptosporidium* sp. * A | *Entamoeba* spp. |  |
| Taeniid * A | *Cystoisospora* sp.  Hookworm  *Ascaris* spp. |  |
| Taeniid * B |  | *Eimeria* spp.  *Sarcocystis* sp.  *Toxocara canis*  *Dipylidium caninum* |
| *Ascaris* spp. * A | *Eimeria* spp.  *Cryptosporidium* sp.  *Toxocara* |  |
